# Cell-Penetrating Peptide and Transferrin Co-Modified Liposomes for Targeted Therapy of Glioma

**DOI:** 10.3390/molecules24193540

**Published:** 2019-09-30

**Authors:** Xi Wang, Yarong Zhao, Shiyan Dong, Robert J. Lee, Dongsheng Yang, Huan Zhang, Lesheng Teng

**Affiliations:** 1 School of Life Sciences, Jilin University, Changchun 130012, China; wangxi1976@126.com (X.W.); zhaoyr16@mails.jlu.edu.cn (Y.Z.); sydong16@mails.jlu.edu.cn (S.D.); 2 Division of Pharmaceutics and Pharmacology, College of Pharmacy, The Ohio State University, Columbus, OH 43210, USA; lee.1339@osu.edu; 3 Department of Chemistry and Pharmacy, Zhuhai College of Jilin University, Zhuhai 519041, China; yds@jluzh.edu.cn

**Keywords:** cell-penetrating peptides, targeting, glioma, blood-brain barrier

## Abstract

Glioma is one of the most aggressive and common malignant brain tumors. Due to the presence of the blood-brain barrier (BBB), the effectiveness of therapeutics is greatly affected. In this work, to develop an efficient anti-glioma drug with targeting and which was able to cross the BBB, cell-penetrating peptides (R8) and transferrin co-modified doxorubicin (DOX)-loaded liposomes (Tf-LPs) were prepared. Tf-LPs possessed a spherical shape and uniform size with 128.64 nm and their ξ-potential was 6.81 mV. Tf-LPs were found to be stable in serum within 48 h. Uptake of Tf-LPs in both U87 and GL261 cells was analyzed by confocal laser scanning microscopy and by flow cytometry. Tf-LPs were efficiently taken up by both U87 and GL261 cells. Moreover, Tf-LPs exhibited sustained-release. The cumulative release of DOX from Tf-LPs reached ~50.0% and showed excellent anti-glioma efficacy. Histology of major organs, including brain, heart, liver, spleen, lungs and kidney, and the bodyweight of mice, all indicated low toxicity of Tf-LPs. In conclusion, Tf-LPs showed great promise as an anti-glioma therapeutic agent.

## 1. Introduction

Glioma is one of the most aggressive and common malignant brain tumors, possessing high growth rate, high recurrence rate and high invasiveness [1,2,3]. The patients with glioma only have a survival rate of less than 10.0% in five years [4]. Surgery and chemotherapeutic agents such as doxorubicin hydrochloride, temozolomide, and paclitaxel have been used in glioma treatment, although only temozolomide was found to be moderately effective [5]. Unfortunately, chemotherapy effects are greatly affected due to the presence of the blood-brain barrier (BBB) [5]. Here, BBB mainly contains two aspects. One is the barrier between brain cells and plasma formed by the cerebral capillary wall and glial cells, the other is the barrier between cerebrospinal fluid and plasma formed by choroid plexus [6]. Most chemotherapeutic agents have difficulty getting across the BBB and entering into the brain, which limits their efficacy [7]. Meanwhile, they also cause toxicity for healthy brain cells and tissues due to their inefficient targeting of glioblastoma. Therefore, the development of an efficient anti-glioma drug with targeting and which is able to cross the BBB is urgently needed.

Nanovehicles represented an attractive strategy for the delivery of chemotherapeutic agents in glioma [8,9]. An ideal nanovehicle for the treatment of glioma should be able to cross the BBB and target tumor cells [10]. In the past few years, various nanocarriers, including polymers and liposomes, had been developed. Liposomes are considered “self” by the immune system and possess many advantages such as good biocompatibility, low toxicity, and non-immunogenicity [11], however, delivery of liposomes across the BBB remains a great challenge [8,9].

Receptor-mediated endocytosis is an important mechanism for targeting and delivery chemotherapeutic agents to the brain [12]. Many efforts had been made to develop the targeted liposomes modified with specific ligands. It has been shown that transferrin receptors (TfRs) are overexpressed in both brain microvascular endothelial cells and glioma cells [13]. If Tf is conjugated to liposomes, glioma cells can be selectively targeted. Yuan et al. [14] synthesized Tf/TAT-PTX/DOX-LP by choosing Tf as the targeting ligand. The result indicated that the targeting efficiency of nanoparticles was improved and excellent results were obtained for melanoma. Wei et al. [13] synthesized TfR-targeting core-shell nanoparticles for delivery of siRNA, showing excellent anti-tumor efficiency [14,15].

Cell-penetrating peptides (CPPs), including TAT, Octa-arginine (R8) have been extensively evaluated in drug delivery [16]. Zong et al. [4] developed dual targeting DOX liposomes conjugated to Tf and cell-penetrating peptide (TAT) for delivery of DOX across BBB and targeting brain glioma, showing an excellent anti-glioma effect. Yuan et al. [14] also prepared liposomes modified with Tf and TAT loading paclitaxel and doxorubicin for therapy of glioma, which enhanced targeting efficiency and increased therapeutic efficacy. Although TAT was effective in the liposomal drug delivery system for therapy of glioma, it has been reported to cause embolization and hemolysis in vivo [4,14,15]. R8, as a short peptide and an alternative to TAT, can be conjugated with a hydrophobic moiety, such as a fatty acid, and incorporated into liposomes [17,18]. Combining Tf and R8 with liposomes can potentially enhance their therapeutic efficiency for glioma.

**Scheme 1:**
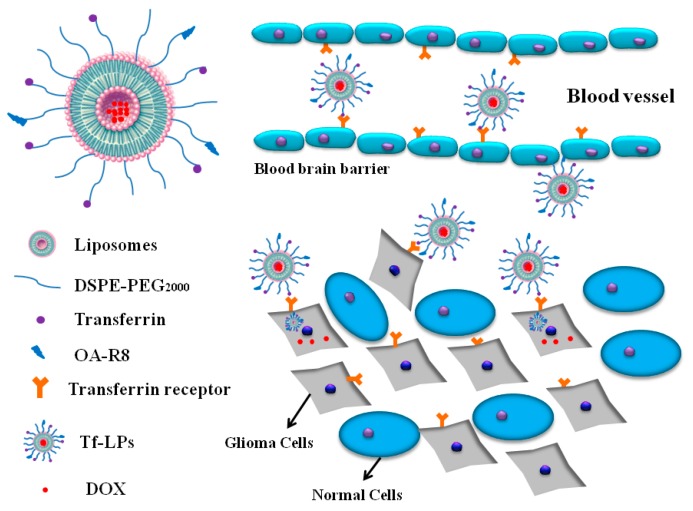
The mechanism for drug delivery to glioma via Tf-modified DOX-loaded liposomes (Tf-LPs). LPs were prepared by the ammonium sulfate gradient method. R8 was used as a cell-penetrating peptide (CPP) to improve the ability of crossing the blood-brain barrier (BBB). Tf was attached to the surface of LPs. Abbreviations: LPs, DOX-loaded liposomes; Tf-LPs, DOX-loaded Tf-liposomes; OA-R8: Oleic acid-modified octa-arginine.

In order to develop an efficient anti-glioma drug with targeting and the ability to cross the BBB, Tf-modified DOX-loaded liposomes (Tf-LPs) were prepared by the ammonium sulfate gradient method and the post-insertion method. R8 was used as a CPP to improve the ability to cross the BBB. In addition, Tf was attached to the surface of LPs, as presented in Scheme 1. Different cationic materials were evaluated to determine the optimum parameters for preparing the LPs. The properties of Tf-LPs including particle size, stability in FBS, encapsulation efficiency and drug release kinetics in vitro were all measured. In addition, cellular uptake of Tf-LPs in vitro was analyzed. Meanwhile, a glioma model was established and used to evaluate the anti-tumor efficacy of Tf-LPs. The biodistribution of Tf-LPs in vivo and histology of organs, including brain, heart, liver, spleen, lungs, and kidney in mice receiving therapy were also investigated.

## 2. Results

### 2.1. Preparation and Characterizations of Tf-Modified DOX-Loaded Liposomes (Tf-LPs)

In order to optimize the formulation of LPs, cationic materials including (2,3-dioleoyl-propyl)- trimethylamine (DOTAP), 1,2-dioleyloxy-3-dimethylaminopropane (DODMA), and dimethyloc- tadecyl ammonium bromide (DDAB) were evaluated, and the results are presented in Table 1. The particle size, polymer dispersion index (PDI), ζ-Potential and encapsulation efficiency were different with different cationic materials. LPs had a mean particle size of 115.2 ± 3.04 nm particle size, PDI of 0.227 ± 0.04, and ζ-potential of 9.64 ± 0.27 mV when DOTAP was used as the cationic lipid. While 153.4 ± 1.85 nm LPs were obtained when DODMA was used, PDI was 0.322 ± 0.09 and ζ-potential was 7.96 ± 0.86. However, when DDAB was used, the particle size was increased to 3568 ± 10.34 nm. Meanwhile, the encapsulation efficiency of LP-1 was greater than LP-2. Therefore, DOTAP was chosen as the cationic material for the preparation of LPs. 

**Figure 1:**
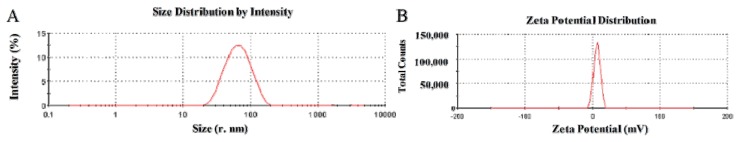
(**A**) the particle size and (**B**) ξ-potential of prepared Tf-LPs. The particle size of Tf-LPs was 128.64 nm. ξ-potential of Tf-LPs was 6.81 mV. Abbreviations: LPs, DOX-loaded liposomes; Tf-LPs, DOX-loaded Tf-liposomes.

Tf-LPs were prepared by the post-insertion method [13]. As shown in Figure 1, the particle size of the obtained Tf-LPs was larger than LPs (115.2 nm) and was 128.64 nm. ξ-potential of Tf-LPs was 6.81 mV, which was lower than that of LPs and the concentration of Tf in Tf-LPs was evaluated by bicinchoninic acid (BCA) protein assay. The concentration of Tf was 16.15 ± 1.28 μg/mL. The conjugation process was found to be reproducible.

**Figure 2:**
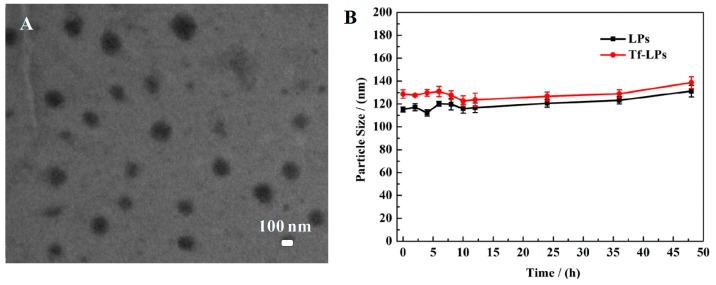
TEM image of Tf-LPs and their colloidal stability in serum (*n* = 3). (**A**) TEM image (×50,000), (**B**) the stability of Tf-LPs in 10% of serum. Tf-LPs possessed spherical shape and uniform size, the diameter was ~90 nm. The stability of Tf-LPs in 10.0% of FBS was measured by DLS, indicating good stability in serum. Abbreviations: LPs, DOX-loaded liposomes; Tf-LPs, DOX-loaded Tf-liposomes.

Tf-LPs were examined under TEM, as shown in Figure 2A. Tf-LPs possessed spherical shape and uniform size (the diameter was ~90 nm), which was consistent with data from DLS. In addition, the stability of Tf-LPs in 10.0% of FBS was measured by DLS, as shown in Figure 2B. The particle size of Tf-LPs within 48 h was relatively unchanged, indicating good stability in serum.

### 2.2. Cellular Uptake In Vitro

**Figure 3:**
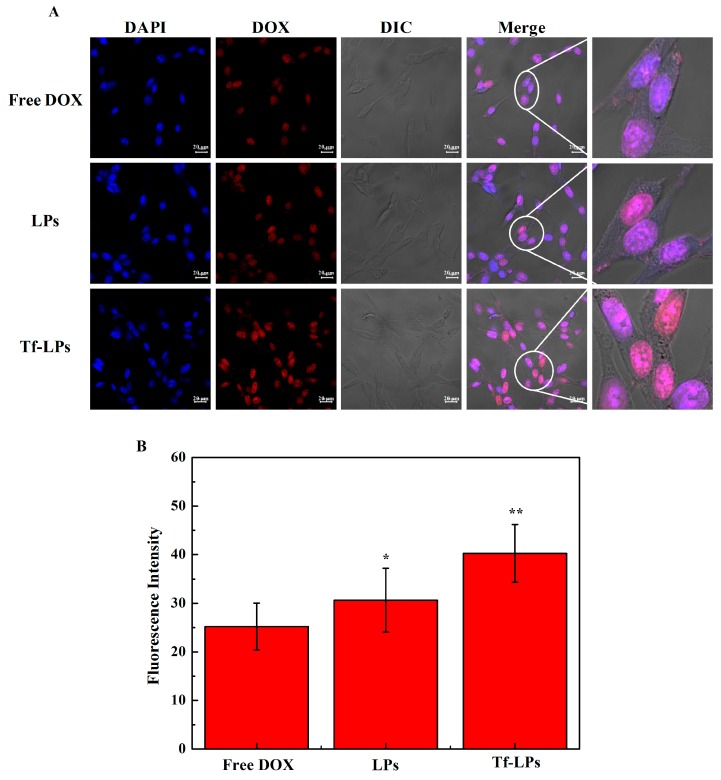
Cellular uptake of Tf-LPs (*n* = 3). (**A**) Confocal laser scanning microscopy (CLSM) images of U87 cells after incubation with free DOX, LPs and Tf-LPs for 4 h, (**B**) the fluorescence intensity of DOX channels when U87 cells were treated with free DOX, LPs and Tf-LPs for 4 h. U87 cells showed the best cellular uptake for Tf-LPs. Abbreviations: * *p* < 0.05, ** *p* < 0.01, LPs, DOX-loaded liposomes; Tf-LPs, DOX-loaded Tf-liposomes.

**Figure 4:**
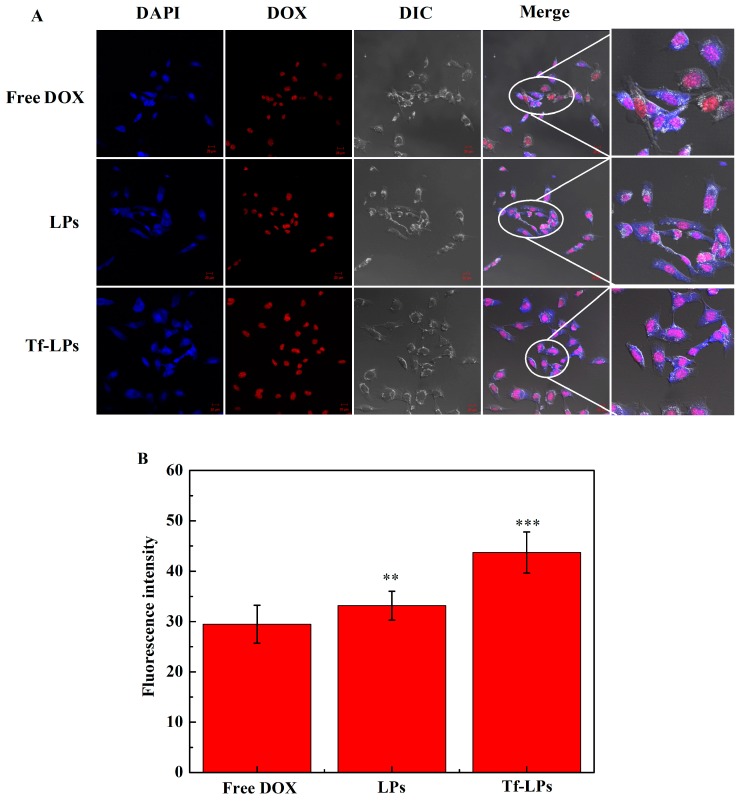
Cellular uptake of Tf-LPs (*n* = 3). (**A**) CLSM images of GL261 cells after incubation with free DOX, LPs and Tf-LPs for 4 h, (**B**) the fluorescence intensity of DOX channels when GL261 cells were treated with free DOX, LPs and Tf-LPs for 4 h. GL261 cells showed the best cellular uptake for Tf-LPs. Abbreviations: ** *p* < 0.01, *** *p* < 0.001, LPs, DOX-loaded liposomes; Tf-LPs, DOX-loaded Tf-liposomes.

Confocal laser scanning microscopy (CLSM) was used to study the cellular uptake of free DOX, LPs, and Tf-LPs, as shown in Figure 3 and Figure 4. Nuclei of U87 or GL261 cell and DOX were observed in blue and red channels, respectively. Free DOX, LPs, and Tf-LPs all showed uptake and the fluorescence intensity of U87 or GL261 cells treated with LPs was stronger than free DOX-treated cells, which indicated that OA-R8 improved the cellular uptake of LPs [11,19]. However, the fluorescence intensity of LPs-treated cells was slightly weaker than Tf-LPs-treated cells. In addition, the fluorescence intensity of DOX channels was quantitatively analyzed by Image J. U87 or GL261 cells were treated with free DOX, LPs, or Tf-LPs for 4 h. The fluorescence intensity of DOX channels of Tf-LPs-treated cells was the strongest, suggesting a targeting effect of Tf [4,20,21]. Moreover, liposomes were localized within nuclei of cells, suggesting that Tf-LPs were indeed taken up by cells and would be beneficial for tumor therapy.

**Figure 5:**
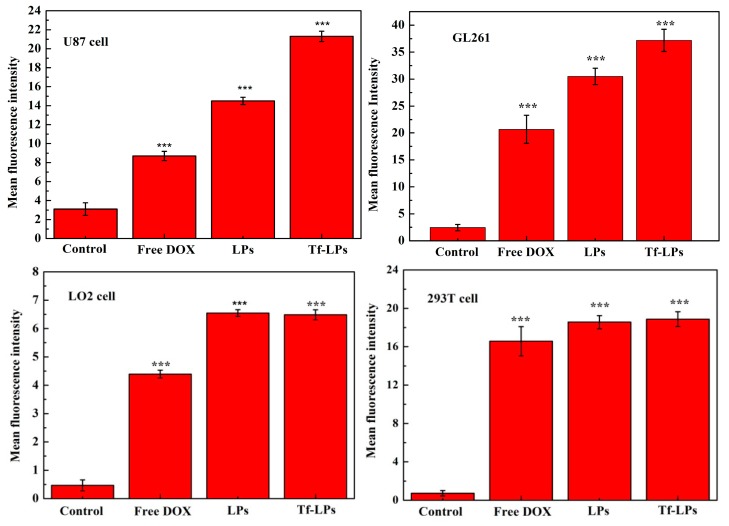
Quantitative analysis of DOX uptake in U87, GL261, LO2, or 293T cells. Cells were treated with free DOX, LPs and Tf-LPs for 4 h (*n* = 3). U87 cells without formulation were used as controls. U87 cells showed the best cellular uptake for Tf-LPs. *** *p* < 0.001, free DOX, LPs and Tf-LPs versus control. Abbreviations: LPs, DOX-loaded liposomes; Tf-LPs, DOX-loaded Tf-liposomes.

Quantitative analysis of cellular uptake was carried out using flow cytometry, as shown in Figure 5. The uptake fluorescence intensity of Tf-LPs was higher than cells treated with free DOX and LPs, while the uptake fluorescence intensity of LPs was greater than free DOX, which indicated that Tf and OA-R8 increased liposomal uptake by U87 and GL261 cells. LO2 cell and 293T cell lines were chosen to evaluate the cellular uptake of TPs and Tf-LPs in normal cells, as shown in Figure 5. The uptake of LPs was higher than free DOX. However, the uptake of Tf-LPs was similar to that of TPs. It can be concluded that Tf-LPs was also taken up by normal cells. Therefore, the formulation parameters including the content of Tf will be optimized in our future work.

### 2.3. DOX Release In Vivo

**Figure 6:**
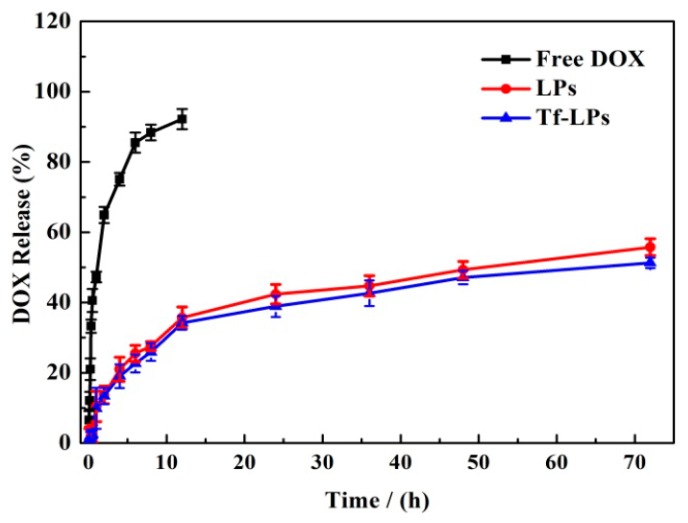
Release kinetics of free DOX, LPs, and Tf-LPs in PBS (pH 7.4, 0.01 M) (*n* = 3). Free DOX was quickly released and the release was close to 90.0%. All DOX-loaded liposomes exhibited sustained release behaviors, ~50% of DOX was released from liposomes. Abbreviations: LPs, DOX-loaded liposomes; Tf-LPs, DOX-loaded Tf-liposomes.

The release properties of DOX from liposomes (LPs and Tf-LPs) were investigated in PBS (pH 7.4, 0.01 M), which are presented in Figure 6. Free DOX was quickly released and the release capacity was close to 90.0%. However, all DOX-loaded liposomes exhibited sustained release behaviors, where ~50% of DOX was released from liposomes. Similar release behavior was observed between LPs and Tf-LPs, implying that the modification with Tf did not affect the release behavior of DOX.

### 2.4. Cytotoxicity Study In Vitro

**Figure 7:**
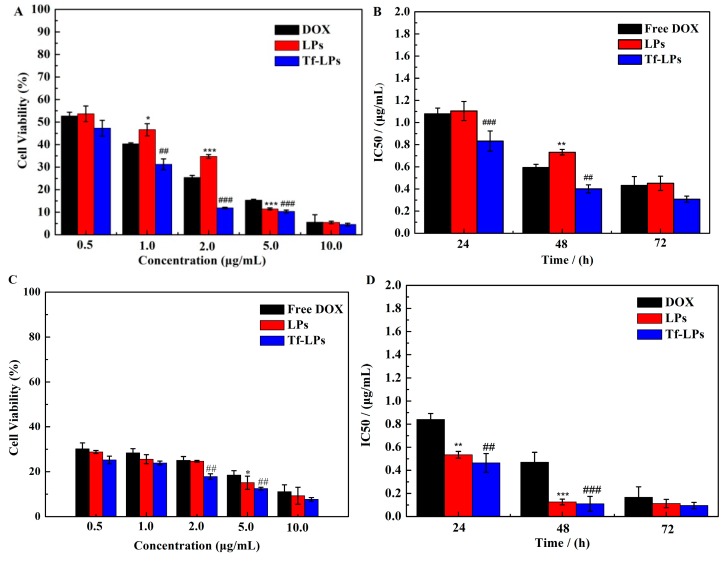
Antitumor activity of free DOX, LPs, and Tf-LPs. (**A**) Viability of U87 cells treated with free DOX, LPs and Tf-LPs for 48 h, (**B**) IC_50_ values of three formulations for U87 cells at 24, 48 and 72 h, (**C**) Viability of GL261 cells treated with free DOX, LPs and Tf-LPs for 48 h, and (**D**) IC_50_ values of three formulations on GL261 cells at 24, 48 and 72 h (*n* = 6). * *p* < 0.05, ** *p* < 0.01, *** *p* < 0.001, LPs versus free DOX; ^##^ *p* < 0.01, ^###^ *p* < 0.001, Tf-LPs versus free DOX. The antitumor activity of Tf-LPs was higher than LPs and free DOX. Abbreviations: LPs, DOX-loaded liposomes; Tf-LPs, DOX-loaded Tf-liposomes.

The antitumor activity of different formulations on U87 cells and GL261 cells was investigated by MTT assay. U87 cells or GL261 cells were treated with free DOX, LPs, or Tf-LPs for 24, 48, or 72 h. As shown in Figure 7A, free DOX presented a higher inhibition rate than LPs on U87 cells at 48 h. Tf-LPs showed stronger anti-proliferation than free DOX and LPs, which indicated that Tf enhanced cellular uptake of Tf-LPs. Moreover, IC_50_ values of free DOX, LPs and Tf-LPs at 24, 48 and 72 h on U87 cells were also calculated, showing a high inhibition rate. The antitumor activity of Tf-LPs was higher than LPs and free DOX. This data further showed that the modification with Tf promoted cellular uptake for DOX and increased the antitumor effect for U87 cells. In addition, GL261 cells were used to investigated the cytotoxic effect of Tf-LPs. Tf-LPs showed stronger anti-proliferative activity than free DOX and LPs. Meanwhile, the activity of Tf-LPs on GL261 cells were higher than that of on U87 cells.

### 2.5. Anti-Glioma Activity In Vivo

**Figure 8:**
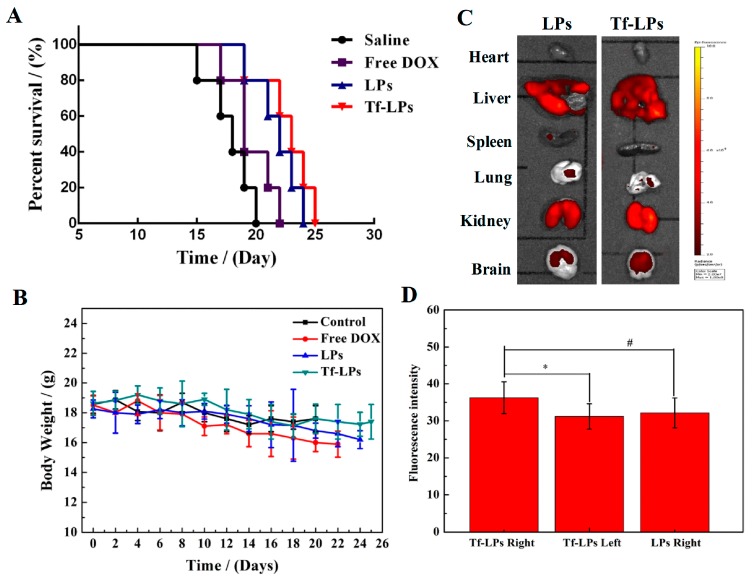
In vivo evaluation of Tf-LPs in U87 murine glioma model (*n* = 3). (**A**) Survival curves and (**B**) the bodyweight of intracranial U87 glioma-bearing mice over time. (**C**) Images of organs after injecting LPs or Tf-LPs for 2 h. (**D**) Quantitative analysis of LPs or Tf-LPs content in the right brain and the left brain. * *p* < 0.05, Tf-LPs right versus Tf-LPs left, ^#^ *p* < 0.05, Tf-LPs right versus LPs right. The median survival time of mice treated with Tf-LPs (25 days) was longer than that of mice treated with saline (20 days), free DOX (22 days) and LPs (24 days). The bodyweight in LPs and Tf-LPs group dropped slower than the free DOX group, suggesting the low toxicity of LPs and Tf-LPs. LPs and Tf-LPs were accumulated in the liver, kidney, and brain. Abbreviations: LPs, DOX-loaded liposomes; Tf-LPs, DOX-loaded Tf-liposomes.

The quality of life and survival time are the major clinical indicators to estimate the effectiveness of antitumor therapy [10]. The anti-glioma efficacy of Tf-LPs was investigated in vivo. The survival curves of U87 glioma-bearing mice at different times after treatment with saline, DOX solution, LPs or Tf-LPs are presented in Figure 8A. The survival time of mice treated with Tf-LPs (25 days) was longer than that of mice treated with saline (20 days), free DOX (22 days) and LPs (24 days), which may be attributed to the presence of cell-penetrating peptide and Tf [20]. These results indicated that liposomes modified with cell-penetrating peptide and Tf exhibited a significant improvement in anti-glioma activity compared to the free DOX solution [4]. In addition, the bodyweight of intracranial U87 glioma-bearing mice at a different time was also measured, as shown in Figure 8B. The bodyweight in the LPs and Tf-LPs groups dropped slower than free DOX group, suggesting the low toxicity of LPs and Tf-LPs.

In order to estimate liposomal delivery to the tumor, major organs of U87 glioma-bearing mice were imaged, as shown in Figure 8C. At 2 h after the injection of DiR-labeled LPs and Tf-LPs, the fluorescence intensity of organs was comparable to each other. Weaker signals were exhibited in the brains of animals treated with TPs and the accumulation of DiR-labeled Tf-LPs was higher, indicating LPs or Tf-LPs have the ability to cross the BBB [4]. In addition, LPs and Tf-LPs were all accumulated in the liver and kidney, this may be because LPs and Tf-LPs were metabolized by kidneys and liver [4]. In addition, LPs or Tf-LPs contents in the right and the left brain were quantitatively analyzed by Image J and the results are presented in Figure 8D. The fluorescence intensity of Tf-LPs in the right brain was stronger than that in left brain and the LP-treated right brain, suggesting enhanced delivery by Tf-LPs. However, targeting of Tf-LPs was not that specific. Tf-LPs were not only distributed in brain tumors but also in normal brain tissue. This was because the TfR was not only highly expressed in U87 cells, but also in normal cells. This was a potential limitation of the Tf-LPs as a glioma-targeting drug delivery system. Therefore, the synthetic parameter of Tf-LPs should be further optimized in future work.

**Figure 9:**
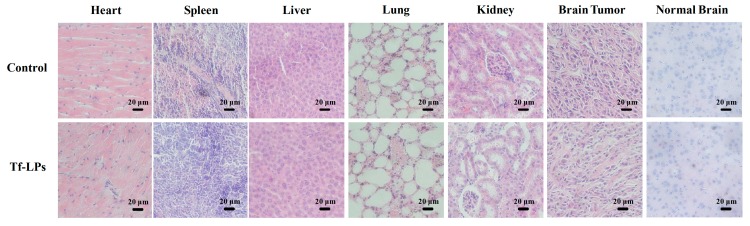
Histological analysis of mice treated with Tf-LPs. Tf-LPs groups showed that no abnormality in these organs were provided, suggesting the safety of Tf-LPs. Abbreviations: LPs, DOX-loaded liposomes; Tf-LPs, DOX-loaded Tf-liposomes.

Meanwhile, to determine the toxicity and treatment effect of Tf-LPs, the Balb/c Nude mice receiving treatment were euthanized and organs were taken and analyzed by H and E staining, as presented in Figure 9. Compared with the control (saline group), the Tf-LPs groups showed that no abnormality in these organs (Heart, Liver, Spleen, Lung, Kidney and Normal brain) were provided, suggesting the safety of Tf-LPs. In addition, the brain tumor treated with Tf-LPs group was significantly better than that of the saline group. It can be concluded that OA-R8 and Tf co-modified liposomes have the ability to cross the BBB and possessed an excellent anti-glioma effect.

## 3. Discussion

Glioma is an incurable disease [1,2,3]. Chemotherapy, as one of the treatments for glioma, is greatly affected due to the presence of the BBB [5]. In this work, CPPs and Tf co-modified DOX-loaded liposomes were prepared to improve trans-BBB delivery. Firstly, LPs were loaded with DOX [22]. It is well known that the surface of the cells possesses abundant negative charges [11]. If cationic materials are added during the preparation of the liposomes, cellular uptake can be increased via electrostatic interactions. Three kinds of cationic materials, including DOTAP, DODMA, and DDAB, were discussed in this work. Different particle size, PDI, and ζ-potential were presented with different cationic materials. However, both particle size and zeta potential are important for the drug delivery system [23,24]. Therefore, DOTAP was chosen as best cationic material for the preparation of LPs.

In order to target glioma cells, Tf was grafted on the surfaces of LPs. It was noticeable that the averaged particle size of Tf-LPs was ~90 nm by TEM, which was smaller than the average size determined by DLS (128.64 nm). This was because DLS showed Tf-LPs in the hydrated state, while TEM displayed particles in the dried state [8]. Both Tf and R8 could improve the cellular uptake of Tf-LPs [11,19]. Due to OA-R8 being able to facilitate liposome uptake by cells, the uptake fluorescence intensity of LPs was higher than that of free DOX. However, the uptake fluorescence intensity of Tf-LPs was similar to that of TPs, this is because the TfR in the normal cells surface is low expressed and because of the absence of the tumor-targeting effect. The only barrier for drug release is the diffusion of the drug from one side to the other of the dialysis membrane in free DOX, whereas, for LPs or Tf-LPs, there are roughly 2 stages: the release of the drug from NPs and then diffusion through the membrane [4,8]. Therefore, the drug release of Tf-LPs presented a slow release rate. For cytotoxicity of Tf-LPs, free DOX showed a higher inhibition rate than LPs at 48 h, the main reason is that free DOX could be quickly transported into cells by passive diffusion under in vitro conditions. However, LPs prevents drug access to tumor cells due to slow-release [4,8,20,21,25]. Due to the existence of Tf, Tf-LPs possessed stronger anti-proliferation than free DOX, indicating that Tf promoted cellular uptake for DOX and increased the antitumor effect. However, Tf-LPs possessed a stronger anti-proliferative effect on GL261 cells than that of U87 cells, this may be attributed to greater sensitivity of mouse glioma for DOX [21,22].

The anti-glioma efficacy of Tf-LPs was also investigated by intracranial U87 glioma-bearing mice in vivo. The survival time of mice treated with Tf-LPs (25 days) was longer than that of mice treated with saline (20 days), free DOX (22 days) and LPs (24 days), this is because Tf could specifically bind to the Tf receptor on the surface of U87 cells and cell-penetrating peptide can facilitate transport across the BBB, further enhancing anti-glioma efficacy [4,20]. Meanwhile, due to the low toxicity of LPs and Tf-LPs, the bodyweight of intracranial U87 glioma-bearing mice decreased slowly. In contrast, free DOX-treated mice had a rapidly decreasing bodyweight, indicating high toxicity [7]. In addition, weaker signals were exhibited in the brains of animals treated with TPs and the accumulation of DiR-labeled Tf-LPs was higher, which is attributed to CPP and Tf [4].

## 4. Materials and Methods 

### 4.1. Materials

Egg phosphatidylcholine (ePC), DOTAP, DODMA, DDAB, transferrin, and 1,2-distearoyl-sn- glycero-3-phosphoethanolamine-*N*-(methoxy-(polyethylene glycol)-2000) (PEG_2000_-DSPE) were all obtained from Avanti Polar Lipids (Shanghai, China). OA-R8 was synthesized by Jill Biochemical Co., Ltd. (Shanghai, China). Cholesterol and DOX were from Sinopharm Chemical Reagent co. Ltd. (Shanghai, China). The U87 cell line was from the American Type Culture Collection (ATCC, Rockefeller, MD, USA). Balb/c Nude mice (male, 6–8 weeks old, 18.0–20.0 g) were obtained from Shanghai Slack Laboratory Animals Co., Ltd. (Shanghai, China). 

### 4.2. Methods

#### 4.2.1. Preparation of DOX-Loaded Tf-Liposomes

LPs were synthesized according to the previously reported method [11,26]. Different cationic materials (DOTAP, DODMA or DDAB,) were evaluated. Firstly, cationic material, OA-R8, ePC, cholesterol and DSPE-PEG_2000_ were dissolved in ethanol at a 20/25/18/32/3 molar ratio to form a mixture. Then, the above mixture was rapidly injected into the ammonium sulfate solution (250 mM) at a volume ratio of 1/10 under rapid stirring. Liposomes were obtained and the ammonium sulfate outside of liposomes was removed by the dialysis method. Finally, DOX was added and actively loaded into liposomes. Excess DOX was removed by ultrafiltration centrifuge tube.

Tf-LPs were prepared by the post-insertion method [27]. Briefly, the products of Tf (20.0 mg) and Traut’s reagent (85.0 μL, 4.0 mg/mL) reacted with Mal-PEG-DSPE (3.6 mg/mL) to obtain Tf-PEG-DSPE under the darkness. After that, LPs were incubated with Tf-PEG-DSPE at 37 °C for 30 min (n_(LPs)_/n_(Tf-PEG-DSPE)_ = 100/1) and Tf-LPs were obtained [13]. Ultrafiltration centrifuge tube with a molecular weight cutoff of 1000 kDa was used to remove excess Tf and the content of Tf was evaluated by a BCA kit.

#### 4.2.2. Characterization of LPs and Tf-LPs

DLS (Nano-ZS ZEN3600, Malvern, UK) was used to characterize the properties of LPs and Tf-LPs, including particle size, PDI and ξ potential. Prior to measurements, 100 μL of LPs or Tf-LPs (lipid concentration is 4.0 mg/mL) were diluted to 1 mL with deionized water. TEM (200 kV, JEOL JEM 2100, Tokyo, Japan) was applied to observe the microstructure of Tf-LPs and the particle size of Tf-LPs was measured by analyzing the digital micrograph. The stability of LPs and Tf-LPs was evaluated in 10% FBS at 37 °C [13,19]. The particle size of LPs and Tf-LPs were measured by DLS at a different time (0, 2, 4, 6, 8, 10, 12, 24, 36 and 48 h). High-Performance Liquid Chromatography (HPLC, LC-20AD, Shimadzu Corporation, Kyoto, Japan) was used to measured DOX concentration. SDS/acetonitrile/methanol(51/42/7, *v*/*v*/*v*) was as the mobile phase. The flow rate was set to 1.0 mL/min and the column temperature was kept at 30 °C. Briefly, LPs or Tf-LPs were diluted in a five-fold volume of methanol and sonicated for 15 min. The suspension was centrifuged at 12,000 rpm for 5 min, the concentration of supernatant was detected. EE was measured by HPLC and calculated by the below equation [8,28]:

$$EE=\frac{m(DOX)}{m(DOX)+m(freeDOX)}\times 100\%$$

where, m(DOX) is the mass of DOX in LPs or Tf-LPs (mg) and m(free DOX) stands for the mass of DOX in the filtrate (mg).

#### 4.2.3. In Vitro DOX Release

DOX release from LPs and Tf-LPs in vitro was studied by dialysis method using PBS (pH 7.4) as the release media [2]. Briefly, a certain amount of free DOX, LPs and Tf-LPs was placed in dialysis bags with the molecule weight cutoff of 12.0 kD. They were immersed in 50.0 mL of release medium and gently stirred in the darkness and the temperature was kept at 37 °C. Then, 1.0 mL of release medium was sampled and 1.0 mL of fresh release medium (37 °C) was supplemented at predetermined time intervals. HPLC was applied to determine the release capacity of DOX from liposomes.

#### 4.2.4. Cell Culture and Uptake In Vitro

U87 (human glioma cells), GL261 (mouse glioma cells), LO2 and 293T cells were cultured in DMEM medium containing 10% FBS, 100 U/mL penicillin and 100 μg/mL streptomycin in 5% CO_2_ at 37 °C [29].

LCSM (Carl Zeiss; Jena, Germany) were applied to qualitatively analyze cellular uptake for LPs and Tf-LPs in vitro [8,25]. Firstly, U87 cells (5 × 10^4^ cells per well) were cultured in a confocal dish for 24 h. Then, free DOX, LPs and Tf-LPs were added to U87 cells and cultured for another 4 h. After that, U87 cells were immobilized with 500 μL of 4% formaldehyde solution and the nucleus was stained for 15 min using DAPI (2.0 μg/mL).

In order to further analyze cellular uptake for LPs and Tf-LPs in vitro, flow cytometry (Coulter Epics XL, Beckman Coulter, California, CA, USA) was used [19]. Briefly, U87, GL261, LO2 or 293T cells (1 × 10^5^ cells per well) were cultured in a 6-well plate for 24 h. After that, free DOX, LPs and Tf-LPs were added into U87, GL261, LO2 or 293T cells and incubated for 4 h. Cells were trypsinized and immobilized with 4% formaldehyde solutions. 

#### 4.2.5. Antitumor Activity In Vitro

The antitumor activity of LPs and Tf-LPs were measured by MTT assay [8]. Firstly, U87 or GL261 cells with 8 × 10^4^ cells per wells were cultured in a 96-well plate for 24 h. Different concentrations (0.5, 1.0, 2.0, 5.0, 10.0 μg/mL) of LPs and Tf-LPs were added to each well and continually incubated for 24, 48 or 72 h. MTT (10.0 μL, 5.0 mg/mL) was added and incubated at 37 °C for 4.0 h. Finally, DMSO was added to dissolve formazan precipitate and OD values were detected by a microplate reader (Synergy4, multi-mode microplate reader, BioTek, Winooski, VT, USA). In this experiment, wells containing U87 or GL261 cells without formulation were regarded as controls.

#### 4.2.6. Glioma Model. Establishment

In order to evaluate the anti-glioma effect of Tf-LPs in human glioma, experiments in vivo were carried out. Firstly, Balb/c Nude mice (male, 6–8 weeks old, 18.0–20.0 g) were anesthetized using 10.0% chloral hydrate (10 μL/10 g dose) and fixed on a stereotaxic instrument [25]. 5.0 μL of U87 cells (the density is 1 × 108 cells/mL) were slowly (the speed is 0.10 μL/min) injected into the right brain of each Balb/c Nude mouse. Finally, these mice were fed for 10 days under the standard condition and used for the next experiments [13]. All animal experiments had been approved by the Animal Ethics Committee of the Jilin University (No. 201805010).

#### 4.2.7. Biodistribution

After the glioma model establishment, the mice were randomly divided into two groups (*n* = 3) [12,30]. PKH26-labeled liposomes and Tf-liposomes were prepared. The same doses (~15.2 μmols phospholipids/kg body weight) of PKH26 labeled liposomes or Tf-liposomes were injected into mice via the tail vein, after 2 h, the organs including brain, heart, liver, spleen, lungs, and kidney were taken to observe the distribution of formulations. Liposome was labeled by PKH26 and the living body excitation light is PKH26.

#### 4.2.8. Anti-Glioma Efficacy

Twenty intracranial mice were divided into four groups where each group consisted of 5 mice. 0.10 mL of Free Dox, LPs, and Tf-LPs with equivalent DOX doses of 2 mg/kg were injected into each mouse via the tail vein every 2 days. The survival times and the weight of each group were recorded every day. In addition, organs of mice including brain, heart, liver, spleen, lungs, and kidney were taken and histological characteristics of these organs were analyzed by H&E staining [7]. The fluorescence intensity of LPs or Tf-LPs in a brain tumor was qualitatively analyzed by Image J.

#### 4.2.9. Statistical Analysis

All data were analyzed by Student’s *t*-test and Log-rank and Wilcoxon tests, and shown as mean ± standard error. 

## 5. Conclusions

In this work, to develop an efficient anti-glioma drug with targeting and the ability to cross the BBB, cell-penetrating peptides (R8) and Tf co-modified DOX-loaded liposomes were prepared. Cellular uptake, cytotoxicity and anti-tumor efficacy of Tf-LPs were evaluated in vitro and in vivo, showing high anti-glioma efficacy and low toxicity. We believe that the data obtained support further development of Tf-LPs as a potential anti-glioma therapeutic agent. In future work, we plan to quantitatively analyze the content of DOX in the brain tumor and evaluate the pharmacokinetics of Tf-LPs in vivo.

## Figures and Tables

**Table 1:** The optimum formulations for preparing LPs (*n* = 3).

Formulation	Cationic Material	Particle Size (nm)	PDI	ζ-Potential (mV)	Encapsulation Efficiency (%)
LPs-1	DOTAP	115.2 ± 3.04	0.227 ± 0.04	14.71 ± 0.27	88.45 ± 1.75
LPs-2	DODMA	153.4 ± 1.85	0.322 ± 0.09	7.96 ± 0.86	75.60 ± 2.47
LPs-3	DDAB	3568 ± 10.34	1.00 ± 0.25	10.7 ± 0.89	/

Abbreviations: LPs, DOX-loaded liposomes; Tf-LPs, DOX-loaded Tf-liposomes; DOTAP, DODMA, DDAB, PDI
