# Correction: Wang et al. Cell-Penetrating Peptide and Transferrin Co-Modified Liposomes for Targeted Therapy of Glioma. *Molecules* 2019, *24*, 3540

**DOI:** 10.3390/molecules27175729

**Published:** 2022-09-05

**Authors:** Xi Wang, Yarong Zhao, Shiyan Dong, Robert J. Lee, Dongsheng Yang, Huan Zhang, Lesheng Teng

**Affiliations:** 1 School of Life Sciences, Jilin University, Changchun 130012, China; 2 Division of Pharmaceutics and Pharmacology, College of Pharmacy, The Ohio State University, Columbus, OH 43210, USA; 3 Department of Chemistry and Pharmacy, Zhuhai College of Jilin University, Zhuhai 519041, China

When reviewing our publications, an error in article [1] caught our attention. The H&E-stained image of the lung for the Tf-LPs treatment (Figure 9) incorrectly used the picture of the lung treated with the control. Here, we provide the corrected Figure 9 below. The data were re-analyzed, and there is no change to the conclusion of the study.

The authors apologize for any inconvenience caused and state that the scientific conclusions are unaffected. This correction was approved by the Academic Editor. The original publication has also been updated.

## Figures and Tables

**Figure 9:**
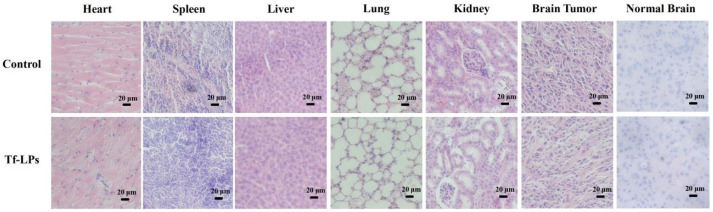
Histological analysis of mice treated with Tf-LPs. Tf-LPs groups showed that no abnormality in these organs were provided, suggesting the safety of Tf-LPs. Abbreviations: LPs, DOX-loaded liposomes; Tf-LPs, DOX-loaded Tf-liposomes.
